# The Bioelectric Code: Reprogramming Cancer and Aging From the Interface of Mechanical and Chemical Microenvironments

**DOI:** 10.3389/fcell.2018.00021

**Published:** 2018-03-06

**Authors:** Brian B. Silver, Celeste M. Nelson

**Affiliations:** ^1^Department of Molecular Biology, Princeton University, Princeton, NJ, United States; ^2^Department of Chemical and Biological Engineering, Princeton University, Princeton, NJ, United States

**Keywords:** mechanotransduction, bioelectricity, morphodynamics, mechanical stress, morphogenesis

## Abstract

Cancer is a complex, heterogeneous group of diseases that can develop through many routes. Broad treatments such as chemotherapy destroy healthy cells in addition to cancerous ones, but more refined strategies that target specific pathways are usually only effective for a limited number of cancer types. This is largely due to the multitude of physiological variables that differ between cells and their surroundings. It is therefore important to understand how nature coordinates these variables into concerted regulation of growth at the tissue scale. The cellular microenvironment might then be manipulated to drive cells toward a desired outcome at the tissue level. One unexpected parameter, cellular membrane voltage (Vm), has been documented to exert control over cellular behavior both in culture and *in vivo*. Manipulating this fundamental cellular property influences a remarkable array of organism-wide patterning events, producing striking outcomes in both tumorigenesis as well as regeneration. These studies suggest that Vm is not only a key intrinsic cellular property, but also an integral part of the microenvironment that acts in both space and time to guide cellular behavior. As a result, there is considerable interest in manipulating Vm both to treat cancer as well as to regenerate organs damaged or deteriorated during aging. However, such manipulations have produced conflicting outcomes experimentally, which poses a substantial barrier to understanding the fundamentals of bioelectrical reprogramming. Here, we summarize these inconsistencies and discuss how the mechanical microenvironment may impact bioelectric regulation.

## Introduction

Membrane voltage (Vm) is defined as the electrical potential difference between the cytoplasm and extracellular space (Levin, 2007). This bioelectric field has been demonstrated to transmit extensive patterning information between cells at tissue-scale. For example, disrupting Vm gradients has been shown to impair regeneration and development, causing the growth of functioning ectopic organs such as eyes in *Xenopus* and head/brain structures in planaria (Beane et al., 2011; Pai et al., 2012). Excitingly, manipulating Vm has also been shown to induce limb regrowth in non-regenerative species (Tseng and Levin, 2013), prompting much interest in bioelectricity as a future therapeutic tool to restore organs deteriorated during the aging process or accidentally damaged. In addition, manipulating Vm can prevent the formation of tumors (Chernet and Levin, 2014), suggesting promising future cancer treatments. However, it is unclear what Vm manipulation is needed to produce a desired outcome; separate studies report contradicting observations resulting from similar alterations of Vm. In particular, comparable Vm manipulations have been linked to both apoptosis and proliferation, which are seemingly opposite phenotypes (Bortner et al., 1997; Wang et al., 1999; Yu et al., 1999a,b; Thompson et al., 2001). Adding another level of complexity, cellular Vm varies significantly between cell types and with progression of the cell cycle. How then might this broad range of observations surrounding Vm be reconciled into a consistent theory for possible implementation in future medical therapies to combat aging and disease?

Although it is generally accepted that experiments in culture do not recapitulate the complexity of the cellular surroundings *in vivo*, a number of parameters altered by traditional culture methods are often not accounted for in experimental design and data interpretation. The ability of chemical components of the cellular microenvironment to impact phenotype is a classic topic of study. For example, factors such as hypoxia (Pang et al., 2016) and pH (Damaghi et al., 2013) have been demonstrated to drive cancer progression. However, it is becoming increasingly well recognized that physical signals also contribute to tumorigenesis: substratum stiffness and pressure are two key components of this *mechanical* microenvironment (Discher et al., 2005; Piotrowski-Daspit et al., 2016). Further studies regarding how mechanical parameters impact Vm are needed to more fully understand the processes that contribute to the emergence of bioelectric field gradients. Additional factors in the microenvironment such as the presence of multiple cell types and microbiota impact the transduction of bioelectric signals (Chernet and Levin, 2014). However, we lack a full understanding of how all of these regulatory cues function together to translate changes in Vm into physiological cellular states.

Not only do variables in the microenvironment change in studies of bioelectricity, but Vm itself may impact several variables within the microenvironment. The conflicting outcomes of previously published experiments then may have resulted from unintentionally altering different regulatory cues simultaneously. In addition, at the cellular level, the output of a given Vm input is often represented as a single biological state, such as growth or death. Apoptosis and proliferation are generally treated as bimodal opposites. However, this interpretation is incomplete at best. Rather than behaving as opposing cellular functions, division and programmed death occur in a coordinated fashion to sculpt growth and form, tuned by the complex web of surrounding microenvironmental signals. This review summarizes the seemingly conflicting reports of bioelectric signaling and discusses two topics that may help explain these inconsistencies: the mechanical microenvironment and the importance of cellular death.

## Bioelectric manipulation: the future of cancer and geriatric therapies?

The idea that electricity could contain biological information was first demonstrated experimentally in the late 1700s, when Luigi Galvani electrically stimulated muscle contraction in an amputated frog leg (Verkhratsky et al., 2006). Although initially met with skepticism, the idea of “animal electricity” (Galvani, 1791) eventually led to our current understanding of how the brain and body are connected. Electrical properties are often only associated with excitable cells, such as neuronal tissue. However, all cells possess an electrical potential across the plasma membrane, and thus generate and receive bioelectric signals (Levin, 2010). One of the earliest indications that this electrical potential might serve as a growth-directing field came in 1903, when A.P. Mathews identified an electrical gradient in regenerating hydra (Mathews, 1903).

**Figure 1:**
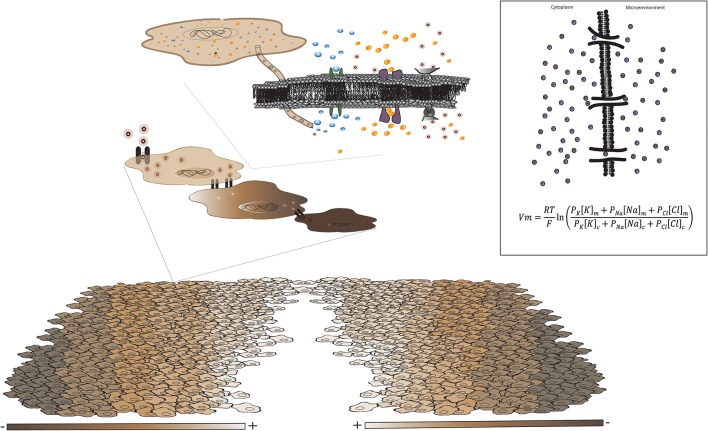
The value of Vm in a given cell is determined by the intra- and extracellular concentrations of all ion species present (predominantly Na^+^, K^+^, and Cl^−^) according to the Hodgkin-Katz-Goldman equation (Binggeli and Weinstein, 1986). Changes to Vm can propagate between neighboring cells via ion exchange through gap junctions, or local voltage gating of ion channels (Levin, 2014). This results in gradients of physiological electric charge within the tissue, referred to as the bioelectric field. For example, a wound creates a depolarized front of migrating cells (Chifflet et al., 2005), acting as a break in the tissue-wide bioelectric circuit.

Modern experiments (Beane et al., 2011, 2013; Pai et al., 2012; Tseng and Levin, 2013; Adams et al., 2016) support the historical hypothesis (Mathews, 1903; Burr and Northrop, 1935) that electrical patterns regulate not only muscle movement and excitable cells as demonstrated by Galvani, but growth and form of the organism as a whole. In the 1970s and 1980s, separate studies measured Vm of different cell types (summarized in Binggeli and Weinstein, 1986). These data showed that cancerous and proliferative tissues were generally more positively charged than non-proliferative cells (Levin, 2012a; Adams and Levin, 2013). Consistent with this observation, it was found that experimentally causing cells to become more negatively charged reversibly blocked cellular proliferation (Cone and Tongier, 1971), which was thought to result from the blockage of ions such as Na^+^ believed to be involved in DNA synthesis (Binggeli and Weinstein, 1986). When the cytoplasm is more positively charged than the extracellular space, Vm is referred to as “depolarized” (Adams and Levin, 2013). Conversely, when the cytoplasm is more negatively charged than the extracellular space, Vm is considered to be “hyperpolarized” (Figure 1). It was therefore hypothesized that a threshold Vm separates “normal” quiescent or resting cells from proliferative or cancerous tissues (Binggeli and Weinstein, 1986). However, this hypothesis did not account for several factors. First, although Vm was measured directly using patch clamping, these measurements were all conducted under different conditions in separate studies. Some measurements were taken directly *in vivo* under live dissection conditions (tumors), others were of single cells cultured on glass (fibroblasts), and some involved tissue slices, explanted organs (corneal epithelium), or intact 4–16 cell-stage embryos (Binggeli and Weinstein, 1986). Notably, it remains unclear how bioelectrical patterns are impacted by age both in cells and whole organisms, adding yet another uncontrolled variable to these data. Even during this early phase of Vm research, it was appreciated that Vm “deteriorates” rapidly under non-physiological conditions: an observation generally not accounted for in reports of that era (Binggeli and Weinstein, 1986). Although cancerous cells were observed to be more depolarized than noncancerous cells (Binggeli and Cameron, 1980), this observation represented an average of the cell population rather than an absolute fixed value.

Today, we recognize that although cellular Vm usually ranges from approximately –90 to –10 millivolts (mV), these values can vary greatly depending on the cell type and physiological state of the cell (Yang and Brackenbury, 2013). Each cell regulates its resting Vm through a variety of mechanisms (Adams and Levin, 2006). ATP-dependent pumps allow the cell to push ions into or out of the cytoplasm, even against their electrochemical gradients. Cells also express a large variety of channels that allow the passage of ions across the plasma membrane. These ion channels may be gated in response to changes in extracellular ion concentrations, Vm, or mechanical stimuli (Coste et al., 2010; Pathak et al., 2014; Wu et al., 2016; Gudipaty et al., 2017). Thus, as might be anticipated, cellular Vm is neither spatially uniform throughout the cell nor static in time. This added level of complexity might carry additional biological signals. For instance, tumor cells confined in narrow microfabricated channels establish a polarized distribution of Na^+^/H^+^ pumps and aquaporins in the cell membrane, creating a net inflow of water and ions at the leading edge of the cell and an outflow at the trailing edge. This flux enables the migration of metastatic breast cancer cells through narrow channels in culture, independent of actomyosin contractility and integrin signaling (Stroka et al., 2014). Inhibiting the Na^+^/H^+^ exchanger involved in this process decreases the velocity of migration. In addition to spatial gradients, temporal variations in intracellular Vm play a significant role in cell cycle progression. Generally, cells are more hyperpolarized during S phase and more depolarized during mitosis, whereas G1 and G2 phases fluctuate partway between these extremes (Barghouth et al., 2015). This appears to be driven by changes in expression levels of ion channels for K^+^, Na^+^, and Cl^−^, and gating of these channels in response to changes in cell volume or alterations in Vm (Ouadid-Ahidouch et al., 2001; Sundelacruz et al., 2009; Urrego et al., 2014; Barghouth et al., 2015). This cyclical variation in Vm observed during the cell cycle is believed to be required for a successful cell division (Barghouth et al., 2015). The regulation of Vm through time would appear then to be a critical part of bioelectrical signaling.

In addition, the role of mechanical factors in regulating both proliferation and Vm is becoming increasingly evident. During the early days of cell culture, an optimal cell density was known to be necessary for the growth of non-immortalized cells: not only did low cell density inhibit growth (few cell-cell contacts), but cells within an overly confluent monolayer also exhibited reduced proliferation (Todaro and Green, 1963). Intriguingly, it was later found that cells in a confluent monolayer are more hyperpolarized than individual cells (Blennerhassett et al., 1989)—an early indication that mechanical forces may help regulate bioelectric signaling. Despite the growing number of observations that Vm is a dynamic property influenced by features of the microenvironment, it is still generally accepted today that dividing or cancerous cells are more depolarized than non-dividing tissue (Wang, 2004; Fraser et al., 2005; Ouadid-Ahidouch and Ahidouch, 2008; Sundelacruz et al., 2009; Yang and Brackenbury, 2013; Chernet and Levin, 2014) or quiescent cells (Barghouth et al., 2015). This concept is supported by recent experiments employing optogenetic control over ion channels, which have demonstrated that hyperpolarization decreases tumor incidence (Sundelacruz et al., 2009; Levin, 2012b; Chernet and Levin, 2014). Such studies generate excitement that bioelectric control could be implemented in medical strategies to combat cancer. This field also holds promise for restoring organs that are damaged or failing due to aging, potentially improving quality of life or even extending lifespan. However, will simply hyperpolarizing a tumor (using drug treatments or optogenetic methods) cause cancerous cells to enter a quiescent state or die? Would depolarizing an area of tissue cause a failing organ to regenerate, or would it cause a tumor to form? The observation that mechanical and chemical factors in the microenvironment interact with Vm signals both spatially and temporally has begun to change the bioelectric view of oncogenesis from a cellular switch in the Vm of healthy cells past a “threshold” value to an organism-wide defect in bioelectric patterning (Levin, 2012b).

## The bioelectric paradox: one input, multiple outputs

Despite the growing attention being given to the promise of bioelectricity in medicine, a consistent theory that connects Vm to a desired phenotype remains largely elusive. Experiments aimed at understanding bioelectric regulation at both the cellular level and during global tissue patterning events such as regeneration have yielded conflicting results. Remarkably, at the cellular level, similar Vm manipulations can trigger both growth and death (Bortner et al., 1997; Wang et al., 1999; Yu et al., 1999a,b; Thompson et al., 2001). Specifically, separate studies in which depolarization was induced in culture using drug treatments or by adjusting ion concentration in the cellular medium reported increases in either proliferation or apoptosis (Magnis et al., 1991; Wang et al., 1999; Wang, 2004; Lang et al., 2005; Yang and Brackenbury, 2013; Leanza et al., 2014; Levin, 2014; Table 1). Further, one paper reports that hyperpolarization by potassium channels is responsible for inhibiting apoptosis of murine myeloblastic FDC-P1 cells, suggesting that increased depolarization is necessary for apoptosis to occur via the Mcl-1 pathway in this cell type (Wang et al., 1999). In contrast, another study reports that hyperpolarization is required for inducing apoptosis, which is thought to occur via an efflux of potassium ions, reducing cell size (Lang et al., 2005). Hyperpolarization drives an influx of calcium ions in some cases (Nilius and Wohlrab, 1992; Ouadid-Ahidouch and Ahidouch, 2008), which has notably been reported to contribute either to apoptosis or proliferation (Clapham, 2007). Although differing levels of calcium ion concentration amplified by Vm changes may seem like an attractive explanation for these disparate observations, it has alternatively been suggested that hyperpolarization does not propel, but inhibits influx of calcium ions by triggering the close of voltage-gated calcium channels (Wang, 2004). It is becoming more widely acknowledged that discrepancies exist regarding what types of ion flow (K^+^, Ca^2+^) contribute to either apoptosis or proliferation (Wang, 2004; Lang et al., 2005). Similar debates persist in studies of global patterning events such as regeneration of amputated limbs or reprograming of oncogenic tissues.

**Table 1:** Apoptosis and proliferation in response to bioelectric field manipulations.

**Experimental system**	**Experimental Vm manipulation**	**Resulting phenotype**	**Reference**
Murine Myeloblastic FDC-P1 cell culture	Depolarization	Apoptosis increased	Wang et al., 1999
Mouse and human lymphoma cells; Jurkat T cells; Mouse cortical neurons	Depolarization	Apoptosis blocked	Bortner et al., 1997; Thompson et al., 2001; Yu et al., 1999a,b
NIH 3T3 fibroblasts	Depolarization	Proliferation blocked	Magnis et al., 1991
*Xenopus laevis*	Hyperpolarization	Inhibition of induced tumor-like structures	Chernet and Levin, 2014; Chernet et al., 2016
*Xenopus laevis*	Misexpression of hyperpolarizing ion channels	Induction of apoptosis or proliferation in the neural tube region, depending on whether dorsal or ventral blastomeres were hyperpolarized, respectively	Pai et al., 2015a
Planaria (*D. japonica*)	Depolarization	Disruption of regeneration: ectopic head formation following bisection;	Beane et al., 2011
*Xenopus laevis*	Hyperpolarization	Ectopic eye formation	Pai et al., 2012

A fundamental question in regenerative medicine is how limbs and organs maintain consistent proportions. A study in planaria determined that depolarization by H,K- ATPase is required for proper head and pharynx scaling following amputation (Beane et al., 2013). Disrupting this Vm gradient resulted in shrunken heads and enlarged pharynxes in regenerated worms. It might be expected that such a phenotype would be caused by defects in proliferation compromising regenerative growth. Surprisingly, apoptotic remodeling of tissues, and not proliferation, was required for proper organ size. Disrupting either depolarization or apoptosis resulted in planaria with disproportionate head and pharynx size in response to amputation. These findings illustrate the link between Vm and apoptosis in regeneration. Although increased depolarization is generally correlated with dividing and cancerous cells (Levin, 2007), in this instance it induced apoptosis during regeneration in planaria.

Still, observations that hyperpolarizing treatments inhibit tumor formation (Chernet and Levin, 2014) might seem to suggest that depolarization is a disease phenotype that potential cancer therapies might seek to abolish. However, artificially creating areas of depolarization in bisected planaria did not generate tumors, but caused the formation of ectopic heads (Beane et al., 2011). Further, artificially hyperpolarizing bisected worms after amputation inhibited normal head regeneration (Beane et al., 2011). These studies indicate that depolarization relative to the surrounding tissue is a critical determinant of normal regenerative processes in planaria. Furthermore, depolarization is required for the regrowth of planarian head structures, including brain and eyes. However, separate studies in *Xenopus* revealed regions of *hyperpolarization* were critical for eye development (Pai et al., 2012). Expression of hyperpolarizing potassium channels induced ectopic eye formation in regions such as the gut and tail, while depolarization inhibited eye formation (Pai et al., 2012). One experiment that may shed light on these seeming discrepancies involved disrupting bioelectric signals by overexpression of hyperpolarizing channels in the frog embryo: widespread apoptosis or proliferation in the central nervous system of the tadpole was observed depending on which cells of the blastula were hyperpolarized, not simply on a specific Vm value (Pai et al., 2015a). This experiment adds an additional level of complexity to our understanding of bioelectricity, illustrating that Vm behaves like a morphogen field (Levin, 2010) rather than a cellular switch. Vm orchestrates behavior at both the cellular (Levin, 2010, 2012b, 2014) and tissue levels (Sundelacruz et al., 2009; Levin, 2010; Adams and Levin, 2013; Chernet and Levin, 2014) in the form of a bioelectric field gradient (Figure 1). In this way, Vm comprises a key component of the external cellular microenvironment. However, in contrast to a traditional chemical morphogen, Vm functions at the interface of chemical and mechanical signals by impacting the flow of biochemically important ions such as Ca^2+^ by creating an electrical gradient across cells. In turn, this electrochemical gradient gates voltage-sensitive ion channels, thus creating a tightly connected communication pathway between the cell and its microenvironment (Clapham, 2007; Ohkubo and Yamazaki, 2012; Rothberg and Rothberg, 2012; Martinac, 2014). In this way, cells can be described as charged points, creating an electrical field across the tissue, termed bioelectricity (Levin, 2007). This bioelectric field is formed by spatial differences in Vm both within and across individual cells. Bioelectricity also carries information temporally, by changing at the same time as growth or injury of the tissue (Levin, 2007, 2010, 2012b, 2014; Tseng and Levin, 2013; Barghouth et al., 2015). For instance, cells located at the edge of a wound become depolarized, then migrate and proliferate to close the lesion (Chifflet et al., 2005). Wounds can thus be described as breaks in the tissue-wide bioelectric circuit (Levin, 2007; Figure 1).

Not only have disparate outcomes been documented in response to bioelectric manipulations, but the postulated mechanism of Vm transduction also differs between studies. Both the planarian and *Xenopus* studies found that calcium signaling was critical for alterations in Vm to be transduced into a morphogenetic output. In planaria, depolarization proceeds via activation of L-type calcium channels, thus increasing the concentration of Ca^2+^ ions in the anterior region of the animal (Beane et al., 2011). Ca^2+^ is then thought to drive anterior gene expression through the activation of factors such as cAMP response element-binding protein (CREB). In *Xenopus*, hyperpolarization-induced ectopic eye formation is also calcium-dependent, as inhibiting voltage-gated calcium (Cav) channels represses this phenotype. *In vivo*, cells reside amongst different cell types as well as in the presence of a complex microbial network. Remarkably, bacteria can participate in propagating Vm signals (Chernet and Levin, 2014). This was discovered in experiments exploring the ability of hyperpolarization to inhibit tumor formation in *Xenopus*. Transduction of this bioelectrical signal into tumor repression proceeds via Vm-modulated transport of histone deacetylase 1 (HDAC1), a factor involved in control of the cell cycle, apoptosis, and differentiation (Chernet and Levin, 2014). Further, HDAC1 was found to be inhibited by butyrate, a by-product of native bacteria in *Xenopus*. In this case, the inhibition of tumor-like structures depends on a balance of both bioelectric cues and microbial (HDAC inhibition) signals, not calcium channels. Therefore, not only do cells internally transduce Vm signals into phenotypic changes, the surrounding microenvironment also plays a substantial role in the physiological outcome of a given Vm input. The key to resolving the differing observations associated with bioelectric manipulation may lie in the consideration of Vm not just as one morphogenetic property, but as a key parameter defined within a network of additional microenvironmental signals.

There are many examples in which disrupting the endogenous bioelectric field, by treating with ion channel inhibitors or overexpressing certain ion channels, has been demonstrated to control organ identity and placement in developing or regenerating organisms (Adams et al., 2007; Levin, 2007, 2010, 2012b; Tseng et al., 2007; Morokuma et al., 2008; Chernet and Levin, 2013; Tseng and Levin, 2013; Barghouth et al., 2015; Neuhof et al., 2016). For instance, depolarization was found to be a key step in the regeneration of the planarian head (Beane et al., 2011). Intriguingly, manipulation of the bioelectric gradient induces regrowth of amputated appendages in species such as *Xenopus* (Tseng and Levin, 2012, 2013), which lose regenerative capability with increasing age. Optogenetic hyperpolarization of amputated tail stumps in *Xenopus* tadpoles was found to induce regeneration of a complete tail structure containing a functioning spinal cord (Adams et al., 2013).

Because manipulating Vm or ion flux can alter phenotypes such as regeneration in aging organisms and correct tissue homeostasis defects such as cancer, much effort is currently being devoted to understanding the gene expression changes through which the bioelectric field regulates regeneration and tissue homeostasis. Specifically, microarray analysis has revealed conserved gene networks regulated by Vm depolarization (Pai et al., 2015b) across three different processes and species (embryogenesis in *Xenopus*; spinal cord regeneration in axolotl; and human cells in culture). Common regulatory processes include cell cycle, cell death, and differentiation, as well as factors associated with cytoskeletal organization, cell interactions, and cell movement. However, these results represent a single time point: further analysis is needed to examine the genetic changes accompanying temporal fluctuations in Vm. An additional study in *Xenopus* revealed that genes associated with hyperpolarization varied temporally in expression (Langlois and Martyniuk, 2013). Specifically, voltage-gated potassium channels decreased at the earliest stages of embryogenesis, but increased in expression during later stages of development. Changes in the levels of these channels could contribute to changes in Vm during growth. This supports the observation that *Xenopus* embryogenesis is accompanied by bioelectric gradients that direct anatomical form (Vandenberg et al., 2011; Adams et al., 2016; Sullivan et al., 2016). Although this information provides valuable insight and confirms that Vm regulation is evolutionarily conserved, it is still unclear how an individual cell translates a given Vm into a fate decision. Furthermore, how is this information coordinated across the cells of a tissue to form the complex structure of an organ? The answers to such questions are collectively referred to as the “bioelectric code” (Tseng and Levin, 2013). Analogous to the way cracking the genetic code provided us with a deeper understanding of heritable illness, understanding the workings of bioelectricity is expected to provide exciting alternative therapies for both cancer and regeneration of organs lost to accidents or deteriorated due to aging, by allowing us to reprogram growth patterns (Levin, 2012b, 2014; Adams and Levin, 2013).

## Aging versus cancer: opposite sides of the same pathology?

There is a strong connection between age and regenerative capacity. For instance, there is an age-dependent decline in the ability of mice to regrow tissues including lung (Paxson et al., 2011) and muscle (Conboy and Rando, 2005). In addition, the proliferative ability of cell populations including β-cells (Tschen et al., 2009) and T-cells (Mackall and Gress, 1997) decreases with age. This decline in regenerative capacity is a large factor in onset of the disability and frailty often associated with aging, due to loss of muscle mass and inability to heal muscle tissue after injury. This is believed to be due to signaling downstream of members of the transforming growth factor beta (TGFβ) family, such as myostatin and growth differentiation factor 11 (GDF11) (Egerman et al., 2015), or Notch (Conboy et al., 2003). However, the mechanism of age-related muscle loss remains unclear. Much effort has focused on identifying molecular components that are impacted by aging: the idea being that artificially returning altered levels of signaling molecules in aged organisms to levels observed in youth might reverse the deleterious effects of aging (Egerman et al., 2015). However, conflicting results have been obtained regarding whether molecules, such as GDF11, are up- or down-regulated as a consequence of aging (Egerman et al., 2015). In addition, simply increasing/decreasing levels of one factor may not be an optimal strategy, as many other processes may be impacted. Tuning the activity of one molecule or pathway without a thorough understanding of all processes that might be impacted could produce unwanted effects. A simple example is that blocking cell death does not provide us with immortality, but instead potentiates the development of cancer (Reed et al., 1996). Bioelectric signaling appears to function as a global regulatory mechanism providing us with the capability of inducing the formation of an entire planarian head without a full understanding of every pathway that is involved in this complex regenerative process (Beane et al., 2011). The ability to regulate regeneration of muscle or deteriorated organs without harming other processes may thus be a potential use of Vm.

Whether changes in bioelectric gradients are involved in the age-related decline of regenerative ability is an open question. More research is needed to determine this. However, Vm may play a role in age-related ailments of neural tissue. In particular, Vm is involved in regulating intracellular Ca^2+^ levels (Clapham, 2007). Ca^2+^ influx occurs through ion channels such as Cav (Catterall, 2011) and transient receptor potential (TRP) channels (Clapham, 2003), while outflux primarily proceeds via uptake into mitochondria and endoplasmic reticulum (ER) (Bezprozvanny and Mattson, 2008). Perturbing this delicate balance can lead to diseases such as Alzheimer's. For example, blocking K^+^ channels might cause a neuronal cell to become more hyperpolarized; this imparts a more overall negative charge to the cell, creating a more favorable electrochemical gradient for entry of positively charged ions (Clapham, 2007; Bezprozvanny and Mattson, 2008) such as Ca^2+^. Elevated intracellular Ca^2+^ levels can lead to increased excitotoxicity and apoptosis (Bezprozvanny and Mattson, 2008), aiding the progression of neurodegenerative disease. It has long been known that a connection exists between Alzheimer's and age (Hardy, 2006). Age is also associated with increased neuronal intracellular Ca^2+^ levels (Thibault and Landfield, 1996). Although Vm and voltage-gated calcium channels are implicated in this process (Thibault and Landfield, 1996), many additional components are involved in heightened Ca^2+^ entry, such as the pores formed by amyloid β-peptide (Bezprozvanny and Mattson, 2008). More research is needed to determine how age impacts bioelectric gradients specifically in both neural and non-neural tissues.

It might be expected that animals with high regenerative capacity are more prone to cancer, because their cells are presumably highly prolific. However, the opposite is true, suggesting that highly regenerative species such as planaria and axolotls have tight regulation of morphogenetic patterning in both space and time (Levin, 2012b). The strong patterning regulation that enables organisms with high regenerative ability not only to maintain their form following injury but to ward off cancerous growth draws a close relationship between cancer and regeneration (Levin, 2012b). Intriguingly, an inverse relationship between the diagnosis of age-related diseases such as Alzheimer's and risk of developing cancer has been observed (Driver et al., 2012). This suggests that cancer, injury, and aging are not necessarily different ailments requiring specialized treatment strategies, but variations of a common morphogenetic patterning defect.

## The apoptotic cell: is information destroyed or does it only change form?

Although there are conflicting results and proposed mechanisms regarding bioelectric signaling, the studies discussed so far appear to agree that Vm transfers some form of information to the cell that is propagated as a stable physiological state (Neuhof et al., 2016). Logically, a change in Vm that triggers division, differentiation, or gene expression in one cell could impact surrounding cells, and thus propagate throughout the tissue. However, changes in Vm can also induce apoptosis. Can a dead cell communicate information to its surrounding tissue? Although counterintuitive, it has become clear that apoptosis is required for many growth and tissue maintenance processes including embryonic development (Haanen and Vermes, 1996), tumor prevention (Hanahan and Weinberg, 2000), and healthy maintenance of the epithelial cell barrier (Rosenblatt et al., 2001; Andrade and Rosenblatt, 2011; Gu et al., 2011; Slattum and Rosenblatt, 2014; Eisenhoffer et al., 2015). The expression of gene networks associated not only with proliferation but also with apoptosis is increased in the first stages of embryogenesis (Langlois and Martyniuk, 2013).

One of the earliest indications that cellular death is necessary for life processes came in 1842, when examinations of amphibian development revealed that both cellular proliferation and death occurred during embryonic growth (Vogt, 1842; Jacobson et al., 1997). Later on, it was recognized that apoptosis is an integral part of many processes during embryogenesis including the formation of the early structure of the brain, which is critical for proper brain function (Oppenheim, 1991; Kuida et al., 1996). Apoptosis plays key roles in sculpting appendages (Saunders et al., 1962; Milligan et al., 1995; Jacobsen et al., 1996), forming tubes and lumina (Glucksmann, 1951), metamorphosis (Lockshin, 1981), controlling cell numbers (Rosenblatt et al., 2001; Andrade and Rosenblatt, 2011), and deleting unwanted structures (Jacobson et al., 1997). Blocking apoptosis in the embryo was found to have deleterious or even fatal outcomes (Kuida et al., 1996). Apoptosis is not only necessary for embryonic development, but is required for regeneration in some species. For example, apoptosis controls the tissue remodeling essential for correct size ratios and cell lineage specification during planarian regeneration (Beane et al., 2013). In addition, studies in *Hydra* revealed that apoptosis was essential for head regeneration following amputation (Chera et al., 2009). Remarkably, a layer of apoptotic cells near the amputation site provided an increased source of Wnt3, functioning in the synchronized division of nearby stem cells. In this way, the dying cells propagated patterning information to the proliferating cells. Not only do apoptotic cells modify the chemical microenvironment of neighboring cells, but their elimination may change the geometry and density of a tissue. Disruption of the homeostatic balance between proliferation and death is proposed to be a primary driving force for both tumorigenesis (proliferation favored over apoptosis) and organ deterioration (apoptosis favored over proliferation) (Andrade and Rosenblatt, 2011; Slattum and Rosenblatt, 2014). The push to understand the disruption of this homeostasis may be behind the unfortunate categorization of apoptosis and proliferation into two opposing phenotypes. However, it is becoming increasingly accepted that apoptosis and proliferation work together to orchestrate growth, development, and maintenance of tissues. Furthermore, the cellular transition from growth to death does not appear to occur as the bimodal switch we often envision. Cells are capable of recovering from apoptosis even after apparently late stages, including caspase activation and DNA damage, a process termed “anastasis” (Tang et al., 2012). During early stages of apoptosis recovery, genes associated with proliferation and cell cycle are enriched. As anastasis progresses, cells take on a migratory phenotype and upregulate genes associated with focal adhesions and regulation of the actin cytoskeleton (Sun et al., 2017). Remarkably, scratch wounds of cell monolayers close faster when induced to undergo anastasis by treatment with and subsequent removal of ethanol, which induces apoptosis (Sun et al., 2017). It is therefore unclear at what point a cell can be considered fully dead, and recovery from apoptosis can even lead to enhanced healing and expression of proliferation-associated genes.

Current strategies for studying bioelectricity often involve manipulating Vm in a given tissue region then observing a particular phenotype, which functions as the output of the Vm input. However, proliferative and apoptotic phenotypes appear to have a complex co-dependence in many situations, and perhaps even cannot be absolutely characterized. The interconnection between apoptosis and proliferation as well as the disparities among bioelectric manipulation experiments imply that the difference between a regenerative signal and a tumor-initiating cue may be very subtle. A more comprehensive understanding of all the factors that contribute to bioelectrical signaling is needed to determine whether a Vm alteration will result in a beneficial or deleterious program.

## Bioelectrical signaling within the mechanical microenvironment

**Figure 2:**
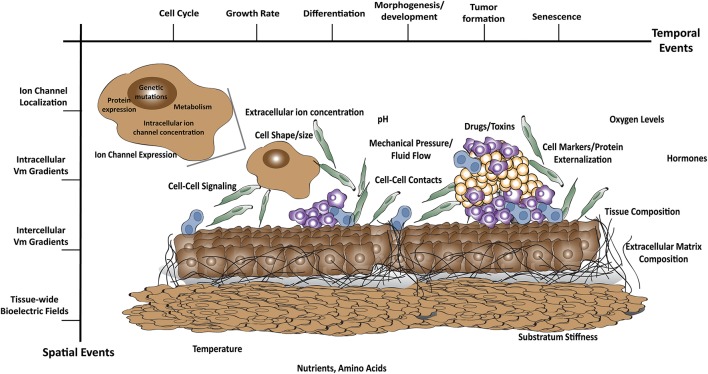
A breadth of factors may impact Vm *in vivo*. All of these factors change not only throughout space (morphogen gradients, mechanical signals) but also over time during growth processes (development, cell cycle, organ regeneration, tumor formation, aging).

Although much attention has traditionally been devoted to understanding how cell-intrinsic parameters (such as genetic alterations and changes in protein expression) drive phenotypes related to aging and cancer, it has become increasingly well recognized that the cellular microenvironment also plays a large role in cancer-related cellular behaviors as well as growth and form at the tissue-scale. The microenvironment functions not only to guide the cell in spatial dimensions, but directs tissue-scale growth through time (Figure 2). In addition, not only is Vm closely related to the microenvironment, the microenvironment is part of the Vm definition. Vm is the difference between electrical charge within the cytoplasm and the external medium (Levin, 2010). Notably, even if the cell did not have any way of controlling its internal charge via ion channels or pumps, Vm could still be changed by altering the charge of the extracellular medium alone.

The involvement of biologically active factors such as pH (Damaghi et al., 2013) in the chemical microenvironment is well recognized. However, it has become increasingly clear that cellular behavior is affected by the mechanical properties of the microenvironment. A number of key properties, including fluid and solid pressure, matrix stiffness (Engler et al., 2006; Kim et al., 2009; Kostic et al., 2009; Tilghman et al., 2010; Zhang et al., 2011; Lee et al., 2012; Pathak et al.), tissue geometry, and mechanical stress (Chen et al., 1997; Dike et al., 1999; Vogel and Sheetz, 2006) comprise the physical microenvironment in a process that depends in part on the mechanosensitive calcium channel Cav3.3 (Walsh et al., 2004; Basson et al., 2015). In addition, pressure activates the oncogenic factors p38, ERK, and c-Src (Walsh et al., 2004). Such findings are of interest because tumors are under higher pressure and also stiffer than the surrounding tissue, creating a microenvironment that promotes cellular proliferation (Basson et al., 2015). Additionally, increased pressure enhances the invasiveness of tumor cells (Piotrowski-Daspit et al., 2016). There are several connections between Vm and mechanical cues. For instance, bioelectric gradients influence osmotic pressure differences *in silico* (Pietak and Levin, 2016). Specifically, hyperpolarization is predicted to lead to lower osmotic pressure than depolarization, due to the outward flow of water predicted to occur along with K^+^ flux out of the cell. Conversely, depolarization is predicted to occur by increased levels of Na^+^ flowing into the cell, where the flow of water is directed from the extracellular space into the cytosol, increasing osmotic pressure.

Substratum stiffness is defined by the amount of force required to deform the surface to which a cell is adhered (Discher et al., 2005). Similar to the way we detect the rigidity of a surface by sensing the amount of force (applied through muscles) required to deform the material, it has been proposed that cells sense the stiffness of their substratum by applying force through actomyosin motors in stress fibers linked to focal adhesions (Kobayashi and Sokabe, 2010). This information is then transmitted to the cell in the form of biochemical signals that direct cellular activities. Varying the stiffness of cellular substrata has been demonstrated to dramatically influence cellular behaviors, including differentiation (Engler et al., 2006), apoptosis (Zhang et al., 2011), proliferation (Tilghman et al., 2010), gene expression (Provenzano et al., 2009; Bordeleau et al., 2015; Cunha et al., 2016), migration (Lo et al., 2000), cell stiffness (Tee et al., 2011), and epithelial-mesenchymal transition (EMT) (Lee et al., 2012). Many of these phenotypes are also regulated by Vm, drawing a tighter possible link between mechanical and bioelectric signaling. In addition, cytosolic Ca^2+^ concentrations play a role in important cancer-related processes including EMT (Davis et al., 2014), metastasis (Prevarskaya et al., 2011), and apoptosis (Orrenius et al., 2003; Zhang et al., 2011). Integrin signaling, a key communication pathway between cells and their ECM, regulates cytosolic Ca^2+^ levels in a manner that depends on both release from intracellular stores as well as influx of extracellular Ca^2+^ through L-type calcium channels (Kwon et al., 2000). This further strengthens the interplay between bioelectrical and mechanical signals. ECM stiffness also regulates mechanosensitive ion channels. For example, Piezo1/2 channels are activated by either stretch or compression (Wu et al., 2016), providing a means through which mechanical signals can be translated into ion flow, which is possibly further propagated toward large-scale bioelectric changes. The importance of mechanosensitive channels is evidenced by their demonstrated role in some cancers (Kobayashi and Sokabe, 2010; Sachs, 2010; Martinac, 2014; Pathak et al., 2014; Li et al., 2015; Xu, 2016).

**Figure 3:**
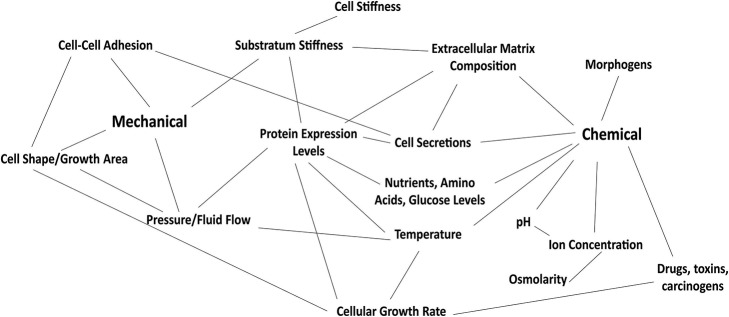
Mechanical and chemical signals present in the cell and surrounding microenvironment are highly interconnected. Altering one variable within this picture could potentially change many factors impacting Vm.

Epithelial cells within a tissue are not only subjected to microenvironments of differing rigidity, but also experience mechanical stress due to the dense packing of neighboring cells. It has been demonstrated that constricting cellular area activates apoptosis programs whereas permitting cellular spreading triggers proliferation (Chen et al., 1997). The physical microenvironment also impacts tissue-level patterning and self-assembly. For example, geometrically constraining endothelial cells on fibronectin-coated strips triggers formation of capillary-like tubes (Dike et al., 1999). During this process, single cells partially detach from the surface to form a hollow central lumen. We still largely do not know how cells sense their position in space and time to go from single cells to the complex multicellular machinery that makes up the body (Vogel and Sheetz, 2006), but the physical microenvironment likely plays a large role. Geometric confinement has also been observed to induce EMT in a manner dependent on ECM stiffness and cytoskeletal dynamics (Nasrollahi and Pathak, 2016). Tissue geometry may also contribute to cancer metastasis. Specifically, cancer cells in culture have been observed to migrate preferentially toward wider branches of microfabricated channels in a manner dependent on cytoskeletal contractility, integrin signaling, and cell alignment along the microchannel walls (Paul et al., 2016). Surprisingly, though, cells confined to narrow channels migrate faster than those in wide channels or on unconstrained surfaces due to increased alignment of stress fibers along the long axis of the channel (Pathak and Kumar, 2012). Mechanical forces are thus postulated to be critical for the prevention of tumor formation (Slattum and Rosenblatt, 2014; Eisenhoffer et al., 2015). This is supported by studies examining the phenomenon of cell extrusion (Eisenhoffer et al., 2015), where cells within a confluent layer are squeezed out in a process that depends on the mechanosensitive ion channel Piezo1. Cell density has been shown to directly influence Vm. Specifically, confluent cells are more hyperpolarized than single cells (Bossu et al., 1992). Cell-cell contacts are critical for propagating bioelectric signals via the transport of ions through gap junctions (Nogi and Levin, 2005; Chernet et al., 2015; Mathews and Levin, 2017). The integrity of cell-cell junctions is altered by mechanical factors including ECM stiffness and culture dimensionality as well as forces from actomyosin contractility, microtubule-based polarization, and integrin/cadherin-dependent adhesion dynamics. Specifically, epithelial cell clusters dissociate more readily on stiffer substrata or when confined to 3D settings (Pathak, 2016). The experimental connections established between chemical, mechanical, and bioelectrical cues place these factors within the same regulatory framework (Figure 3). However, the ways through which the bioelectric field may interact with the mechanical microenvironment and the consequent implications are still unclear.

Traditionally, cells are cultured in single layers on plastic dishes at low confluency. Although observing single cells spread on a plastic substratum may seem like an ideal opportunity to isolate and observe the intrinsic properties of a given cell, nature usually does not subject cells to the conditions recommended by conventional culture techniques. For example, the measured elastic modulus of mammary epithelial tissue is on the order of 150 Pa (Paszek et al., 2005) while that of polystyrene is on the order of 10^9^ Pa (Paszek et al., 2005; Gilbert et al., 2010), over ten million times greater. This realization has led to the question of how closely experiments performed in culture and *ex vivo* can be compared to conditions *in vivo* (Paszek et al., 2005; Gilbert et al., 2010). This question is of great relevance because there are indications that as cells experience the passage of time, they are imprinted with a “memory” of their surroundings.

## Bioelectrical regulation: sending a signal or recalling a memory?

Of course, we are all familiar with the concept that our brain translates experiences in our environment into bioelectrical signals (action potentials) and changes in physical structure (connectivity) between neurons allowing us to preserve the memory of an event (Bailey and Kandel, 1993). The retention of this experiential information from one time point to the next guides future behavior. Although a discussion of memory (not to be confused with the concepts of higher-level thought or consciousness) is often restricted to neuroscience, analogous processes occur in many contexts within non-excitable cells. As with neuronal tissue, non-excitable cells also transduce cues from their surroundings into information (Neuhof et al., 2016). Several levels of information are encoded by cells in the form of stable physiological states that guide cellular behavior. These include genetic sequences, epigenetic factors (histone modifications, DNA methylation), metabolic differences, protein expression levels, and Vm (Neuhof et al., 2016). Cellular memory will be defined here simply as the transfer of such information from one time point to a future one, guiding subsequent cellular activities (Neuhof et al., 2016).

The Weismann barrier refers to a postulate that arose in the early stages of evolutionary science, which dictates that information can only be transferred from germ cells to somatic cells, not in reverse (Weismann, 1893). This would prevent a skin carcinoma that arose from a mutation in the DNA encoding for p53 in epidermal cells exposed to ultraviolet light (Brash et al., 1991) from being passed down to offspring. However, a germline mutation in p53 (Li-Fraumeni syndrome) resulting in increased cancer risk would be a heritable trait (Malkin et al., 1990). This theory was intended to explain why acquired traits did not appear to be transmissible. However, it is becoming recognized that some mechanisms may violate this postulate, such as epigenetic modifications. It has been noted that the incomplete erasure of DNA methylation patterns during germ cell development may result in the transfer of genetic modifications from the soma to germ cells (Hajkova et al., 2002). The mechanical microenvironment may provide another important route via which the Weismann barrier can be circumvented. Oocytes are derived from germ cells, providing half the nuclear genetic material as well as the majority of the membrane and cytoplasm required for reproduction (Li and Albertini, 2013). Intriguingly, the maturation and development of germ cells is controlled by somatic cells. Follicular somatic cells directly contact the oocyte throughout growth, maturation, and fertilization of the egg (Buccione et al., 1990). The somatic cells, therefore, not only transmit chemical signals, but also play a role in transforming the mechanical microenvironment of the germline cells. Mechanical factors within the cellular microenvironment are one means of information transfer between cells (Yang et al., 2014). For example, substratum stiffness directs lineage specification during the differentiation of mesenchymal stem cells (Engler et al., 2006; Yang et al., 2014). In this way, the stem cells preserve a “memory” of their previous ECM stiffness, in the form of a biological lineage. Migrating cells also preserve a memory of past ECM stiffness. Epithelial cells traveling from a stiff to a soft substratum migrate faster and form larger focal adhesions than cells traveling from soft to stiff, even 3 days after they arrive on the soft surface. This mechanical memory depends on nuclear localization of YAP (Nasrollahi et al., 2017). However, YAP is not the only mechanism of mechanical memory. MiRNA-21 levels gradually adjust to ECM stiffness, remaining stable for days after the cells transfer to the new substratum. This process was found to be responsible for stiffness-mediated regulation of fibrosis in mesenchymal stem cells. Either culturing cells on soft ECM or decreasing the levels of miRNA-21 to “erase” cellular memory of stiff ECM was found to protect against fibrosis, scarring, and pro-inflammatory responses in stem cell transplantation experiments (Li et al., 2017). This finding may increase the success of stem cell therapies for tissue repair in damaged or deteriorated organs. In addition to impacting tissue repair and cancer progression, the mechanical microenvironment plays a role in sculpting growth during embryogenesis. Fluid flow is involved in shaping branched tissues in the developing embryo such as vasculature and airways (Nelson and Gleghorn, 2012). In this way, mechanical information might also be transferred from the maternal microenvironment directly to the embryo, without necessarily being mediated by germ cells.

The ways that mechanical information from the microenvironment impact bioelectricity are not fully understood. Intriguingly, gap junctional communication between somatic and germ cells is essential for growth (Li and Albertini, 2013). Gap junctions are physical channels between two cells that allow the passage of small molecules and ions (Alexander and Goldberg, 2003). Gap junctional communication is therefore an important route to propagate bioelectrical signals (Nogi and Levin, 2005; Levin, 2014; Chernet et al., 2015; Mathews and Levin, 2017). Bioelectrical signaling patterns have been observed to be a key part of development and embryogenesis. For example, patterns of depolarization induced by H^+^-V-ATPase were found to be necessary for proper left-right patterning in Xenopus embryos; disrupting the bioelectric field with drug treatments that increase depolarization causes heterotaxia (Adams et al., 2006). Another study found that optogenetically disrupting Vm in only the outermost ectodermal layers in the frog blastula was sufficient to induce craniofacial abnormalities (Adams et al., 2016). Proper patterns of bioelectricity are also required for correct development of the central nervous system in *Xenopus* (Pai et al., 2012). The importance of Vm in embryogenesis raises the question of which direction bioelectric information travels during development: is Vm an intrinsic signaling code emitted from cells during growth, or a physiological memory *imprinted* on cells by their surroundings? A better understanding of how the microenvironment contributes to Vm may enable us to more accurately recapitulate bioelectric patterns at will.

## Untangling the direction of information flow: decoupling the mechanical microenvironment and bioelectrical signaling

Computational network analysis is an increasingly necessary tool in biology (Ma'ayan, 2011) due to the vast number of variables involved in physiological systems. Modeling tools from neuroscience applications may also be useful for understanding electrical dynamics in nonexcitable cells (Pezzulo and Levin, 2016). This will require that we know what parameters to model. Toward this end, a better understanding of how mechanical parameters in the microenvironment impact Vm is needed. One of the key concepts computational studies seek to illuminate is the idea of self-organization of a morphogenetic field, or “symmetry-breaking” of an initially homogeneous state (Pietak and Levin, 2016). Although often discussed in the context of embryonic development (Levin, 2005), mechanisms of asymmetry emergence are also important for understanding how patterning fields become disrupted during the onset of pathologies such as cancer. The generation of heterogeneous patterning cues is thought to occur largely through positive feedback mechanisms that amplify small variations from the realm of noise into measurable signals (Pietak and Levin, 2016). Therefore, even small interactions between the chemical and mechanical microenvironments with local Vm states may play a substantial role in the establishment of global bioelectric regulatory fields. Models such as BETSE (BioElectric Tissue Simulation Engine) examine computationally the emergence of Vm steady states (Pietak and Levin, 2016). The BETSE model considers parameters such as extra/intracellular ion concentration, membrane permeability, cell-cell junctions, and positive feedback between these factors. However, this system has several limitations: specifically, division/apoptosis, mobility including galvanotaxic movement, intracellular Vm components such as the mitochondria/ER, and control of ion channel gene expression are not considered. Furthermore, many additional feedback mechanisms may exist *in vivo*, where not only chemical properties such as ion concentrations and morphogens are at work, but also physical factors such as pressure, stiffness, and geometrical constraints.

Many of the experiments aimed at understanding bioelectric signaling employ input/output-based strategies, where endogenous Vm is altered, and the resulting phenotypic change observed. However, one single regulatory cue does not function in isolation: Vm responds and communicates with the cellular microenvironment via several feedback loops (Pietak and Levin, 2016). For example, many ion channels are themselves gated by changes in Vm; in turn, the ion influx or efflux alters Vm, and the voltage-gated ion channels continue responding to these fluctuations. Changes in expression levels of ion channels can theoretically impact the amount of ion flux occurring in response to physiological triggers such as Vm alterations. Since a number of ion channels are upregulated in tumorigenic cells, one possible treatment idea being explored is ion channel inhibition or knockdown (Li and Xiong, 2011; Stock and Schwab, 2015). However, simply targeting individual ion channels may not be an ideal strategy to combat cancer. First, not all tumors express the same ion channel targets (Schönherr, 2005). Second, Vm is established by ion channels that are gated posttranslationally. As a result, two cells that are in the exact same genetic and transcriptional states could theoretically be in very different bioelectrical states (Levin, 2014). Conversely, the identity of the ion channel is less important, as two cells with very different ion channel transcriptional profiles may be in the same bioelectric state. Bioelectrical signaling can thus be missed by conventional mRNA and genetic profiling. Third, there is substantial redundancy among ion channels: knocking down a single ion channel might not change Vm because other channels with similar function may be triggered to upregulate their activity in response to the knockdown (Levin, 2014). This phenomenon is referred to as ion channel compensation.

Overall, the major limitation of these strategies is that we cannot yet fully say what phenotypic changes, if any, will arise from simply blocking an ion channel or inducing hyperpolarization in a region of tissue. Influencing one variable such as Vm could have broad impacts on many aspects of the cellular microenvironment. For example, Vm depolarization decreases cellular stiffness (Callies et al., 2011). Therefore, a depolarizing treatment that reduces the stiffness of one cell could theoretically alter the mechanical stiffness experienced by a neighboring cell. It is therefore not surprising that bioelectric field manipulations have been documented to produce a wide variety of sometimes inconsistent phenotypes depending on the organism under study and the experimental setup. More studies are needed to fully decouple the outcomes of bioelectrical and mechanical signaling. Toward that end, further research examining the impact of specific changes in the physical microenvironment, including substratum stiffness, cellular stiffness, pressure, geometrical constraint, cell density, cell types, and dimensionality on bioelectrical signaling may help lead to an understanding of the events that cause symmetry breaking and self-generation of morphogenetic patterning events. In addition, although it is known that bioelectrical fields change dynamically during development, it is unclear how Vm signals are altered by the passage of time during aging. The ability of Vm manipulations to regenerate organs such as eyes and limbs has exciting implications for organ restoration in aging individuals, but a more thorough understanding of the long-term effects of Vm manipulations is critical to the success of such a procedure. Toward this end, additional studies monitoring the impact of Vm changes through time in adult organisms would be of great benefit.

## Author contributions

All authors listed have made a substantial, direct and intellectual contribution to the work, and approved it for publication.

### Conflict of interest statement

The authors declare that the research was conducted in the absence of any commercial or financial relationships that could be construed as a potential conflict of interest.

## Abbreviations

ECM: extracellular matrix
HDAC: histone deacetylase
Vm: membrane voltage.
